# Bile Acids in Neurodegenerative Disorders

**DOI:** 10.3389/fnagi.2016.00263

**Published:** 2016-11-22

**Authors:** Hayley D. Ackerman, Glenn S. Gerhard

**Affiliations:** Department of Medical Genetics and Molecular Biochemistry, The Lewis Katz School of Medicine at Temple UniversityPhiladelphia, PA, USA

**Keywords:** bile acids, neuroprotection, neurodegeneration

## Abstract

Bile acids, a structurally related group of molecules derived from cholesterol, have a long history as therapeutic agents in medicine, from treatment for primarily ocular diseases in ancient Chinese medicine to modern day use as approved drugs for certain liver diseases. Despite evidence supporting a neuroprotective role in a diverse spectrum of age-related neurodegenerative disorders, including several small pilot clinical trials, little is known about their molecular mechanisms or their physiological roles in the nervous system. We review the data reported for their use as treatments for neurodegenerative diseases and their underlying molecular basis. While data from cellular and animal models and clinical trials support potential efficacy to treat a variety of neurodegenerative disorders, the relevant bile acids, their origin, and the precise molecular mechanism(s) by which they confer neuroprotection are not known delaying translation to the clinical setting.

## Background

Bile acids are a structurally related group of molecules derived from cholesterol that are widely known for their role as chemical detergents involved in the intestinal absorption and transport of fats and lipid-soluble nutrients (Schonewille et al., 2016). However, bile acids also appear to play as yet poorly defined physiological roles in the central nervous system (Lieu et al., 2014). Surprisingly little work has been done on the physiological roles of bile acids in neurons or the central nervous system (Zhang et al., 1997) despite a wide array of data in model systems and despite the significant therapeutic advantages of bile acids. Bile acids are readily bioavailable via oral, subcutaneous, or intravenous administration, can cross the blood-brain barrier, are relatively nontoxic, and have been approved by the U.S. Food and Drug Administration for human therapeutic use. We review evidence supporting a potentially therapeutic role for bile acids in a number of diverse neurodegenerative conditions. A summary of the studies described below is presented in Tables 1, 2.

**Table 1:** **Model system and human data implicating bile acids in neurodegenerative disorders**.

**Disease/Phenotype**	**Model**	**Bile acid**	**Mode of administration**	**Dose or concentration**	**Effects**	**References**
Spinocerebellar ataxia type 1 (SCA1)	Transgenic mouse with human ATXN1 with 82 CAG trinucleotide repeats under control of a Purkinje-cell promoter	TUDCA	Subcutaneous injection	500 mg/kg	TUDCA crossed the blood-brain barrier but had no effect on cell survival	Kaemmerer et al., 2001
Huntington's disease (HD)	3-nitropropionic acid-treated rat neuronal RN33B cells	TUDCA	Media	100 μM	Decreased apoptosis by preventing mitochondrial depolarization and outer membrane disruption	Rodrigues et al., 2000
		GUDCA		500 μM		
		UDCA		500 μM		
HD	3-nitropropionic acid-treated rats	TUDCA	Intraperitoneal injection	50 mg/kg	Decreased apoptosis	Keene et al., 2001
					Reduced lesion volume	
					Preserved sensorimotor and cognitive function	
HD	R6/2 transgenic mouse with 150 CAG trinucleotide repeats in exon 1 of the huntingtin gene	TUDCA	Subcutaneous injection	500 mg/kg	Reduced striatal cell apoptosis	Keene et al., 2002
					Decreased levels of intracellular inclusions	
					Improved locomotor and sensorimotor function	
Parkinson's disease (PD)	Sodium nitroprusside-treated human dopaminergic SH-SY5Y cells	UDCA	Media	50–200 μM	Dose dependent inhibition of apoptosis via the PI3K-	Chun and Low, 2012
					Akt/PKB pathways	
					Reduced ROS and reactive nitrogen species	
					Maintained intracellular GSH levels	
PD	Skin fibroblasts from PD patients	UDCA	Media	10–100 nM	Restored mitochondrial function dependent upon glucocorticoid receptor activation and Akt phosphorylation	Mortiboys et al., 2013
PD	MPTP-induced mouse model	TUDCA	Intraperitoneal injection	50 mg/kg	Reduction in loss of dopaminergic neurons by preserving levels of phosphorylated JNK, reducing ROS levels, and activating the Akt pathway	Castro-Caldas et al., 2012
Alzheimer's disease (AD)	Aβ-treated primary rat cortical neurons	TUDCA	Media	100 μM	Decreased nuclear fragmentation and cytochrome c release through the PI3K pathway	Solá et al., 2003
AD	Aβ-treated mouse BV-2 microglial cells	UDCA	Media	250 μM	Anti-inflammatory effect by inhibiting NF-κB activation	Joo et al., 2004
AD	Aβ-treated primary rat cortical neurons	UDCA TUDCA	Media	100 μM	Decreased apoptosis dependent on interaction with the mineralocorticoid receptor	Sola et al., 2006
AD	Double transgenic APP/PS1 mice	TUDCA	Diet	0.4% wt/wt	Reduced amyloid plaque number	Nunes et al., 2012; Ramalho et al., 2013
					Decreased injury to neurons	
					Improved memory retention	
					Decreased the loss of a postsynaptic marker in the hippocampus	
Amyotrophic lateral sclerosis (ALS)	Mouse NSC-34 cells with the human SOD1^G93A^ mutation	GUDCA	Media	50 μM	Decreased cell death by blocking caspase-9 activation	Vaz et al., 2015
ALS	Primary mouse ventral midbrain cultures	CA	Media	10 μM	Increased neuronal survival and promoted neurogenesis via LXR	Theofilopoulos et al., 2013
ALS	Clinical trial with ALS patients	UDCA	Oral	Up to 50 mg/kg	UDCA is well tolerated and well absorbed by oral administration	Parry et al., 2010
					UDCA crosses the blood-brain barrier in a dose-dependent manner	
ALS	Clinical trial with ALS patients	UDCA	Oral	3.5 g/140 mL/day	Slight decrease in progression of ALS but results were inconclusive	Min et al., 2012
ALS	Clinical trial with ALS patients	TUDCA	Oral	1 g, twice daily	TUDCA is well tolerated	Elia et al., 2016
					Treatment resulted in improved function and slowed disease progression	
Prion disease	Prion infected male mice	UDCA	Diet	0.01% wt/wt	Reduced astrogliosis	Cortez et al., 2015
					Prolonged survival	
Cerebrotendinous xanthomatosis (CTX)	Human patients	CDCA	Oral	15 mg/kg/day	Amelioration of neurological symptoms	Bjorkhem, 2013
					Improved prognosis	
Retinitis pigmentosa (RP)	Homozygous P23H rhodopsin transgenic rats	TUDCA	Intraperitoneal injection	500 mg/kg	Reduced photoreceptor loss	Fernandez-Sanchez et al., 2011
					Preserved structure, function, and synaptic contacts of rods and cones	
RP	Transgenic *rd10* mouse model	TUDCA	Subcutaneous injection	500 mg/kg	Higher cone cell density from decreased apoptosis Preserved structure and function of photoreceptor cells	Boatright et al., 2006; Phillips et al., 2008; Oveson et al., 2011
Light-induced retinal degeneration	Light-induced retinal damage (LIRD) mouse model	TUDCA	Subcutaneous injection	500 mg/kg	Higher cone cell density from decreased apoptosis Preserved structure and function of photoreceptor cells	Boatright et al., 2006; Phillips et al., 2008; Oveson et al., 2011
Leber congenital amaurosis	Homozygous LRAT knockout mice	TUDCA	Subcutaneous injection	500 mg/kg	Slowed cone degeneration in the ventral and central retina by preventing apoptosis and increasing ER-associated protein degradation	Zhang et al., 2012; Fu and Zhang, 2014
*In vitro* retinal degeneration	Whole mount cat retinas	TUDCA	Media	0.5 μM	Greater receptive field size	Xia et al., 2015
					Decreased irradiance threshold	
					Maintenance of the contrast threshold	
Retinal dystrophy	Primary human retinal epithelium cells	TUDCA	Media	100 μM	Protective against H_2_O_2_-induced impairment of phagocytosis	Murase et al., 2015
Retinal detachment	Subretinal injection of hyaluronic acid in rats	TUDCA	Intraperitoneal injection	500 mg/kg	Reduced apoptosis in the outer nuclear layer of the retina	Mantopoulos et al., 2011
					Decreased caspase activation and protein carbonyl production	
Diabetic retinopathy	Primary rat retinal neuron cells exposed to elevated glucose	TUDCA	Media	100 μM	Decreased apoptosis	Gaspar et al., 2013
					Decreased mito-nuclear translocation of apoptosis-inducing factor (AIF)	
					Decreased ROS and protein carbonyl production	
Retinal ganglion cell degeneration	Intravitreal injection of NMDA in rats	TUDCA	Intraperitoneal injection	500 mg/kg	Increased survival of retinal ganglion cells	Gomez-Vicente et al., 2015
Ischemic stroke	Middle cerebral artery occlusion in rats	TUDCA	Intravenous injection	400 mg/kg	Reduction in infarct size	Rodrigues et al., 2002
					Reduced apoptosis	
					Preserved mitochondrial integrity	
Hemorrhagic stroke	Collagenase	TUDCA	Intra-arterial injection	200 mg/kg	Decreased lesion volumes, peri-hematoma apoptosis, caspase activity, NF-κB activiation; increased AKT activation, neurobehavioral improvement	Rodrigues et al., 2003
Acute neuroinflammation	Intracerebro-ventricular injection with LPS in mice	TUDCA	Intraperitoneal injection	500 mg/kg	Reduced glial cell activation	Yanguas-Casás et al., 2014
Acute neuroinflammation	Primary cultures of microglial cells and astrocytes from rats treated with LPS and/or IFN-γ	TUDCA	Media	200 μM	Reduced microglial chemotaxis and expression of MCP-1 and VCAM-1	Yanguas-Casás et al., 2014
Sleep-wake pattern	Wild type and histamine deficient mice	UDCA	Diet	32 mg/kg	Promotes wakefulness through disinhibition of the histaminergic system via GABA_A_ receptors	Yanovsky et al., 2012
Hypothalamic network activity	Primary cultures of mouse posterior hypothalamus	CA	Media	Up to 8 mM	Reduced firing, synchronized network activity, and blocked GABA_A_ and NMDA receptor activity	Schubring et al., 2012
		GCA				
		TCA				
		DCA				
		TDCA				
		CDCA				
		GCDCA				
		TCDCA				
		DHCA				
Neurotransmitter release	Sympathetic ganglion neurons isolated from of adult bull frogs	CA	Media	1 μM	Inhibits N-type calcium channel currents	Lee et al., 2012
Hyperbilirubinemia	Unconjugated bilirubin-treated organotypic-cultured hippocampal slices from rats	GUDCA	Media	50 mM	Decreased cell death, NOS, glutamate release	Silva et al., 2012
Glutamate-induced neurotoxicity	Glutamate-treated primary rat cortical neurons	TUDCA	Media	100 μM	Decreased apoptosis by activating a PI3K-dependent Bad signaling pathway	Castro et al., 2004

**Table 2:** **Genomic and metabolomics data implicating bile acids in neurodegenerative disorders**.

**Disease**	**Approach**	**Genetic association**	**References**
PD	Meta-analysis of GWAS data from PD and normal patients	HSD3B7 missense SNP in HSD3B7,	Cheng et al., 2003; Song and Lee, 2013
PD	Meta-analysis of PD miRNA GWAS data	SNPs in a miRNA-binding site in the 3' UTR of HSD3B7	Ghanbari et al., 2016
ALS	Peripheral blood cell eQTL of ALS and normal patients	CYP27A1 eQTL	Diekstra et al., 2012
AD	Plasma metabolomic analysis of AD and normal patients	Increased plasma GUDCA levels in patients with mild cognitive impairment or AD	Mapstone et al., 2014

## Biology of bile acids

Two aspects of bile acid metabolism are relevant to their role in neurodegenerative disorders, bile acids that circulate systemically and that are synthesized by neurons. Circulating bile acids are largely synthesized from cholesterol in the liver (Prawitt et al., 2011). Ingestion of food causes bile acid secretion from the gallbladder through the common bile duct to the duodenum in order to facilitate the absorption of lipids and lipid-soluble vitamins via formation of micelles. Upon reaching the ileum, bile acids are transported by specific transport proteins to the portal circulation for recycling back to the liver. The process is highly efficient with over 95% of bile acids resorbed, the remaining 5% proceeding to the colon and excreted through the stool. Enterohepatic recycling of the bile acid pool occurs about 12 times per day, thus the net flux of bile acids through primarily the portal, but also the systemic, circulation is substantial.

The two primary bile acids produced by the liver in humans are cholic acid (CA) and chenodeoxycholic acid (CDCA). These primary bile acids can undergo conjugation with glycine or taurine prior to secretion in the bile to form glycocholic acid (GCA), taurocholic acid (TCA), glycochenodeoxycholic acid (GCDCA), and taurochenodeoxycholic acid (TCDCA). In the intestine, they can undergo dehydroxylation by gut bacteria to produce deoxycholic acid (DCA) and lithocholic acid (LCA). Further chemical modifications can also occur resulting in other minor species such as ursodeoxycholic acid (UDCA) (Zhang et al., 1997).

**Figure 1:**
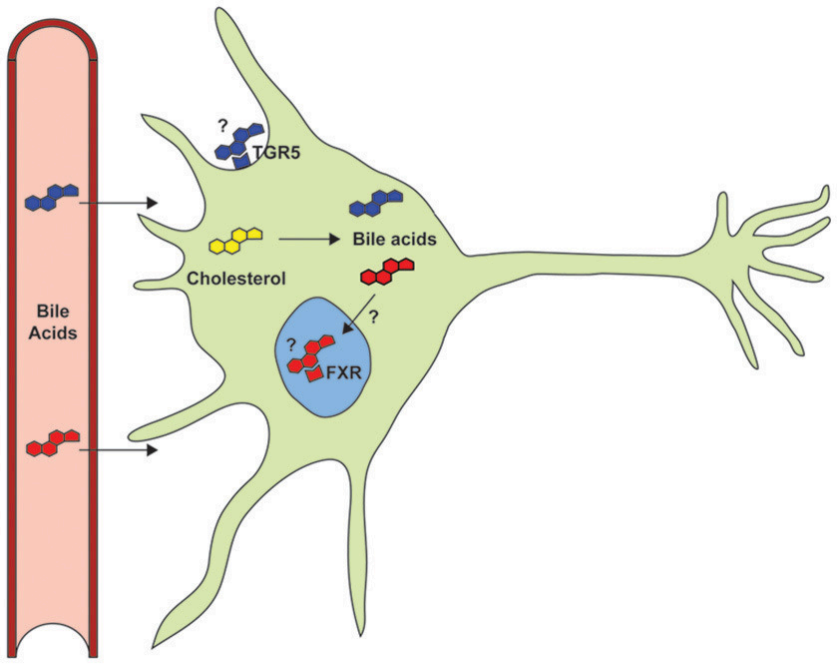
**Despite documented neuroprotective roles in models of neurodegenerative disorders, the primary signaling pathways (TGR5 and FXR) and the potential role of endogenous bile acids have not yet been studied**.

Bile acids also function as signaling molecules through interaction with several receptor systems (Figure 1). They serve as ligands for the nuclear transcription factor farnesoid X receptor (FXR), which forms a heterodimeric complex with retinoid X receptor α (RXRα) that binds to an inverted repeat sequence in gene promoters (Hoeke et al., 2014). They are also agonists for a cellular receptor, the G protein-coupled bile acid receptor 1 (GPBAR1 or TGR5), to mediate signaling via the generation of cyclic adenosine monophosphate (cAMP) by adenylate cyclase, which stimulates cAMP-dependent protein kinase A (PKA) and phosphorylation of the cAMP response element binding protein (CREB) transcription factor (Hodge and Nunez, 2016; Schonewille et al., 2016).

## Alzheimer's disease

Alzheimer's disease (AD) is characterized by dementia and memory loss that is associated with extracellular senile plaques of amyloid precursor protein derived amyloid-beta (Aβ) protein and intracellular neurofibrillary tangles consisting of the Tau protein (Scheltens et al., 2016). A primary *in vitro* model of AD is therefore treatment of cells with Aβ peptide that causes cytotoxicity to both primary cultures of neurons as well as neuronal cell lines. Treatment of primary cortical neuron cultures derived from Wistar rat embryos with tauroursodeoxycholic acid (TUDCA) prior to exposure to Aβ peptide significantly decreased nuclear fragmentation and cytochrome c release that was dependent upon activation of the PI3K pathway (Solá et al., 2003). The bile acid UDCA was found to regulate IκBα and NF-κB regulated genes in the mechanism of protection against Aβ toxicity in the BV-2 microglial cell line (Joo et al., 2004).

Certain steroid hormones exhibit neuroprotective properties that are thought to be mediated in part through interaction with nuclear steroid receptors, including the glucocorticoid, and mineralocorticoid receptors (Garcia-Segura and Balthazart, 2009). Neurosteroids and bile acids are cholesterol derivatives that share some structural similarity so certain bile acids are natural ligands for these receptors. UDCA and TUDCA reduced Aβ induced apoptosis of primary rat cortical neurons that was found to be dependent upon the mineralocorticoid but not the glucocorticoid receptor (Sola et al., 2006). TUDCA appeared to interact with the ligand binding domain of the mineralocorticoid receptor to prevent its binding to heat shock protein 90 and subsequent trafficking for proteosomal processing allowing for translocation to the nucleus.

Bile acids have also been studied in animal models of AD. In double transgenic mice (APP/PS1) that express human amyloid precursor protein carrying the KM670/671NL Swedish double mutation and the human presenilin 1 L166P mutation under the regulation of a neuron-specific promoter, treatment via dietary supplementation of standard laboratory chow with 0.4% (wt/wt) of TUDCA significantly reduced amyloid plaque number in the frontal cortex and hippocampus, decreased injury to neurons measured by determining loss of, or damage to, neuronal fibers surrounding plaques, and improved memory retention measured via contextual, though not auditory, fear conditioning (Nunes et al., 2012) as well as reduced hippocampal and prefrontal amyloid deposition (Lo et al., 2013). TUDCA has also been shown to help preserve the postsynaptic marker postsynaptic density-95 in the hippocampus of APP/PS1 mice (Ramalho et al., 2013). Repurposed RXR agonists or “rexinoids” have also shown some effectiveness in transgenic models of AD (Koster et al., 2016), suggesting a possible involvement of bile acids.

In humans, a recent study of cognitively intact patients identified and validated a set of blood-based biomarkers that included glycoursodeoxycholic acid (GUDCA) that could predict the onset of either AD or amnestic mild cognitive impairment, considered an early precursor of AD, within 2–3 years with an accuracy of over 90% (Mapstone et al., 2014). This suggests a potential association of bile acids in the progression or preclinical neurodegenerative phase of AD.

## Parkinson's disease

The effects of bile acids on chemical and genetic models of Parkinson's disease (PD), which is characterized by the selective loss of dopaminergic neurons in the substantia nigra region of the brain and a resulting tremor, have been reported. Sodium nitroprusside (SNP)-induced cytotoxicity of human dopaminergic SH-SY5Y cells has been used as a model of PD. UDCA was found to dose-dependently (50–200 μM) decrease SNP-related cell death. UDCA reduced reactive oxygen species (ROS), reactive nitrogen species (peroxynitrite and nitric oxide), and helped to maintain intracellular glutathione (GSH) levels. Apoptosis markers including nuclear fragmentation, caspase activation, and cytochrome c release were correspondingly reduced. Inhibiting phosphatidylinositiol-3-kinase (PI3K) and Akt/PKB blocked the favorable effects of UDCA on SNP-induced cytotoxic cell death (Chun and Low, 2012).

Bile acids have also been implicated in PD through genetic association studies. In a meta-analysis of genome-wide association study (GWAS) data based on the genotypes of 2,525,705 SNPs in 4238 PD cases and 4239 non-PD controls, a total of 3 SNPs were found to be statistically associated with PD, including a non-synonymous missense variant in HSD3B7 (hydroxy-delta-5-steroid dehydrogenase, 3 beta- and steroid delta-isomerase 7) (Song and Lee, 2013). Recessive mutations in HSD3B7, which catalyzes the second step in the classical pathway of bile acid synthesis, are associated with loss of bile acid synthetic capability and progressive liver disease (Cheng et al., 2003). In a GWAS of 48,844 SNPs residing in miRNA-binding site variants, 32 SNPs were associated with PD that were located in the 3′ untranslated regions of 13 genes including HSD3B7 (Ghanbari et al., 2016), providing further genetic evidence for a role of bile acids in PD.

Mitochondrial dysfunction has been associated with PD (Luo et al., 2015). To identify compounds that could restore mitochondrial function in skin fibroblasts obtained from patients with a PD parkin (*PARK2*) gene mutation, a 2000 compound library was screened for significant improvement in mitochondrial membrane potential (Mortiboys et al., 2013). Ursocholanic acid and the related compound dehydro(11,12)ursolic acid lactone were among the top 15 compounds that had dose response characteristics favorable for drug development and lacked many of the disadvantages of the other top hits. The structurally related bile acid UDCA was also found to rescue mitochondrial function to a similar extent, which was dependent upon activation of the glucocorticoid receptor and increased phosphorylation of Akt. UDCA was also found to restore mitochondrial function in fibroblasts obtained from a PD patient with a LRRK2-G2019S mutation.

The 1-methyl-4-phenyl-1,2,3,6-tetrahydropyridine (MPTP) neurotoxin is a widely used toxin model for PD, replicating most of the clinical and pathological features of PD in humans and animal models. TUDCA was found to play a role in ameliorating neurodegeneration in MPTP-induced degeneration of dopaminergic neurons in the nigrostriatal axis in C57BL/6 glutathione S-transferase pi (GSTP) null mice (Castro-Caldas et al., 2012). Treatment of mice with TUDCA prior to MPTP caused a 30% reduction in loss of dopaminergic neurons and reduced dopaminergic fiber loss. MPTP toxicity has also been associated with increased ROS production and activation of JNK-mediated apoptosis (Huang et al., 2016). TUDCA reduced levels of ROS and preserved levels of phosphorylated JNK (p-JNK) (Castro-Caldas et al., 2012).

## Amyotrophic lateral sclerosis

The loss of motor neurons in Amyotrophic lateral sclerosis (ALS) has prompted therapeutic strategies aimed at preventing neuronal cell death and promoting regeneration (Carvalho et al., 2015). NSC-34 cells, created by fusion of cultured neuroblastoma cells and motor neurons from mouse spinal cord, can be treated with retinoic acid to induce neurite outgrowth and functional characteristics of motor neurons and are considered a highly stable and widely used murine motor neuron cell line model (Maier et al., 2013; Veyrat-Durebex et al., 2014). NSC-34 cells modified to carry the human form of SOD1 with the G93A mutation (hSOD1^G93A^) have thus been used as a model for ALS. GUDCA was reported to reduce cell death in the NSC-34 hSOD1^G93A^ cell model and to block caspase-9 activation (Vaz et al., 2015).

Bile acids also appear to have physiological roles in the central nervous system. CA, a bile acid present in the adult brain, was identified by LC/MS as a ligand of liver X receptor (LXR), activating LXR but not FXR in ventral midbrain dopaminergic neurons (Theofilopoulos et al., 2013). 6α-hydroxylated bile acids and the synthetic bile acid ligand GW3965 were also highly potent activators of LXR. Male LXR knockout mice develop an adult-onset motor neuron degeneration that is associated with impairment of motor coordination, axonal atrophy, astrogliosis, accumulation of lipid and loss of motor neurons in the spinal cord, findings similar to the neuropathology of ALS (Andersson et al., 2005). Additionally, FXR has been shown to contribute to normal motor function in mice (Huang et al., 2015).

Several clinical trials of bile acids have been conducted in ALS patients in which evidence for safety and potential efficacy was observed (Parry et al., 2010; Min et al., 2012; Elia et al., 2016). In an ALS clinical trial with orally administered UDCA, the bile acid was found to be well tolerated and crossed into the cerebrospinal fluid in a dose-dependent manner (Parry et al., 2010). In a separate clinical trial to test the efficacy of UDCA for treating ALS patients, oral solubilized administration for 3 months was shown to be well tolerated and there was a slight decrease in the progression of ALS in the treatment group as compared to the placebo group (Min et al., 2012). However, due to the small size of the study and the high rate of patient dropout, the efficacy of the treatment was inconclusive. TUDCA administered orally twice per day for over 1 year resulted in a higher percentage of subjects achieving at least a 15% improvement in the ALS Functional Rating Scale Revised (ALSFRS-R) slope (Elia et al., 2016).

Genetic data also implicates bile acid metabolism in ALS. Gene expression profiles obtained from the peripheral blood cells of sporadic ALS patients and normal controls were analyzed in the context of genome-wide SNP genotype data to identify expression quantitative trait loci (eQTLs). A cluster of transcript-SNP pairs with the highest level of statistical significance and meeting correction for multiple testing was associated with *CYP27A1* expression in ALS (Diekstra et al., 2012). CYP27A1 is a key enzyme in the alternative bile acid synthesis pathway, and mutations in this enzyme can cause cerebrotendinous xanthomatosis as described below.

## Huntington's disease

Huntington's disease (HD), an autosomal dominantly inherited neurodegenerative disease, is caused by an expansion mutation in CAG triplet repeat number in exon 1 of the huntingtin (*HTT*) gene (Ross et al., 2014). It is characterized by involuntary choreic movements, psychiatric and behavioral disturbances, and impaired cognitive function. Genetic and chemical animal and cellular models have been developed in which the effects of bile acids have been assessed. For example, 3-nitropropionic acid, an irreversible chemical inhibitor of mitochondrial succinate dehydrogenase, can be used to induce apoptosis of cells in culture and in neurons in the striatum region of the brain that causes changes similar to the morphology and neurochemical changes of HD. Incubation of rat neuronal RN33B cells with TUDCA was found to reduce 3-nitropropionic acid induced apoptosis by about 80% (Rodrigues et al., 2000). Other bile acids, including UDCA and GUDCA, were also associated with decreased apoptosis, preventing 3-nitropropionic acid induced release of mitochondrial cytochrome c, and associated morphological changes in both isolated mitochondria and in intact cells. In rats treated with TUDCA, a similar level of reduction in apoptosis and in the volume of lesions associated with administration of 3-nitropropionic acid was observed that was correlated with preservation of sensorimotor function and performance in cognitive task assays (Keene et al., 2001). These results suggest that the mechanisms of bile acid protection may be similar both *in vitro* and *in vivo*.

Genetic models of HD have also been studied, including the R6/2 mouse, which is transgenic for a 150 repeat trinucleotide CAG expansion in exon 1 of *HTT* and manifests severe neurodegeneration, neuronal intranuclear inclusions, and sensorimotor deficits that result in a severely shortened survival of less than 4 months of age (Mangiarini et al., 1996; Davies et al., 1997). TUDCA treatment was administered to the R6/2 transgenic mice subcutaneously at a dose of 500 mg/kg once every 3 days from weaning to 6 months of age, which was tolerated without skin reaction or other effects. The subcutaneously administered TUDCA was absorbed into the circulation, crossed the blood-brain barrier, and produced seven-fold increases in UDCA levels in the brain without evidence of adverse effects. TUDCA treatment resulted in significantly reduced striatal cell apoptosis, decreased levels of intracellular inclusions, and improved locomotor open field and sensorimotor Rota-Rod performances (Keene et al., 2002).

## Spinocerebellar ataxia type 1

Spinocerebellar ataxia type 1 (SCA1) is another dominantly inherited neurodegenerative disorder characterized chiefly by progressive ataxia (Meera et al., 2016). SCA1 is caused by expansion of an unstable CAG trinucleotide repeat that encodes a glutamine tract in the Ataxin-1 gene (*ATXN1*). The disorder is characterized primarily by degeneration of Purkinje neurons of the cerebellum. TUDCA treatment was used to determine whether the survival of Purkinje cells and the onset and progression of ataxia was altered in a transgenic mouse model of SCA1, in which 30 copies of the human *ATXN1* cDNA containing a CAG trinucleotide repeat of 82 repeats was under the control of the Pcp-2 Purkinje-cell specific promoter l (Kaemmerer et al., 2001). Despite the successful administration of TUDCA using a similar subcutaneous injection protocol as described above for the R6/2 transgenic HD mouse, and documentation of increased levels in the brain, no effects on cell survival or on the neurological phenotype were noted. The disparate effects in two different genetic models of neurodegenerative disease, suggest that bile acids target specific pathways.

## Prion diseases

Neurodegenerative disorders caused by prions include Creutzfeldt-Jakob disease and similar disorders in animals such as bovine spongiform encephalopathy in cows, chronic wasting disease in deer, and scrapie in sheep (Imran and Mahmood, 2011; Windl and Dawson, 2012). Prion protein (PrP^C^) is a normal cellular protein that when mutated becomes misfolded (PrP^Sc^). PrP^Sc^ can then convert normally folded PrP^C^ to the misfolded form. Accumulation of PrP^Sc^ causes loss of neurons, astrogliosis, and spongiform degeneration resulting in dementia, ataxia, and death. One approach to therapeutic development has been to block or interfere with the conversion of PrP^C^ to PrP^Sc^. TUDCA and UDCA were found to substantially reduce this conversion in cell-free aggregation assays as well as in both chronically and acutely infected mouse ScN2a neuroblastoma cells (Cortez et al., 2015). TUDCA and UDCA also reduced neuronal loss in a prion organotypic slice culture model of intracerebral infection that assesses prion replication occurring *ex vivo* through infection of brain slices with prion infected brain homogenate. UDCA treatment also reduced astrocytosis and prolonged survival in prion infected male C57BL/6 mice, although whether bile acids interacted with the PrP^C^ to PrP^Sc^ conversion or mediated protective effects through some other mechanism is not known.

## Cerebrotendinous xanthomatosis

Cerebrotendinous xanthomatosis (CTX) is a very rare autosomal recessive disorder caused by mutations in the *CYP27A1* gene (Björkhem and Hansson, 2010; Bjorkhem, 2013). These mutations lead to deficiency of the sterol 27-hydroxylase inner mitochondrial membrane protein. Sterol 27-hydroxylase oxidizes cholesterol to 27-hydroxycholesterol in the alternative bile acid synthesis pathway that leads to the generation of CDCA. Sterol 27-hydroxylase deficiency leads to a reduction of CDCA and upregulation of cholesterol 7α-hydroxylase (CYP7A1), the rate-limiting enzyme in the classic bile acid synthesis pathway resulting in elevated levels of cholestanol and bile alcohols. CTX patients have a mean age of diagnosis of 35 years and manifest multiple neurologic symptoms including dementia, ataxia, peripheral neuropathy, epilepsy, myopathy, and psychiatric disorders, as well as a variety of non-neurological manifestations including premature atherosclerosis, tendon xanthomas, juvenile onset cataracts, osteoporosis, and mild pulmonary insufficiency. Long-term treatment with CDCA can result in amelioration of neurological symptoms and an improved prognosis.

## Retinal diseases

Bear bile has been used in Chinese medicine for several millennia to treat visual disorders (Boatright et al., 2006). This has led to a number of studies investigating the primary constituent of bear bile, TUDCA, as a potential therapeutic agent for several ophthalmological diseases (Boatright et al., 2009). TUDCA has been investigated in retinitis pigmentosa, a heterogeneous group of disorders of retinal degeneration in which progressive peripheral and night vision loss occurs, with central vision impairment. Mutations in about 200 genes, including the gene encoding rhodopsin (RHO), have been identified that cause apoptosis of photoreceptor cells (Daiger et al., 2013). The RHO P23H mutation is the most common cause of retinitis pigmentosa in the United States that is thought to produce structurally altered folding with retention in the endoplasmic reticulum and resulting cytotoxicity. TUDCA administered intraperitoneally once a week from weaning until 4 months of age to homozygous P23H line-3 rats reduced photoreceptor loss across the retina and preserved synapses between photoreceptors and bipolar or horizontal cells (Fernandez-Sanchez et al., 2011). In the *rd10* mouse model of retinitis pigmentosa, in which the mice carry a missense mutation in exon 7 of the *Pde6b* gene resulting in rod photoreceptor cell death within a month after birth, subcutaneous administration of TUDCA resulted in higher cone cell density in all 4 quadrants of the retina (Oveson et al., 2011). TUDCA also decreased apoptosis, preserved the normal retinal photoreceptor cellular architecture, and maintained amplitudes of dark-adapted electroretinogram a- and b-waves (Boatright et al., 2006), even at later stages of severe photoreceptor loss (Phillips et al., 2008).

Mutations in the retinoid isomerase (*RPE65*) or lecithin-retinol acyltransferase (*LRAT*) genes underlie Leber congenital amaurosis (den Hollander et al., 2008). LRAT is involved in 11-cis retinal recycling in the retinal pigment epithelium through mediating the esterification of all-trans retinol to all-trans retinyl esters, which are the substrates for RPE65. Homozygous *Lrat* knockout mice that were subcutaneously administered TUDCA every 3 days from 1 to 4 weeks after birth had about a three-fold increase in cone density in the ventral and central retina and increased ER-associated protein degradation (Zhang et al., 2012; Fu and Zhang, 2014). Twice per week subcutaneous injections of TUDCA also preserved ERG b-waves and the outer nuclear layer of the retina in Bardet-Biedl syndrome type 1 mice (*Bbs1*^M390R/M390R^), another model for retinal degeneration (Drack et al., 2012).

TUDCA ameliorated cell death and loss of photoreceptor function after exposure to high levels of light (10,000 lux) that can induce retinal degeneration in albino Balb/C mice (Boatright et al., 2006). TUDCA pre-treatment of *ex vivo* perfused whole mount feline retinas repeatedly exposed to light stimulation and dark adaptations over 5 h resulted in greater receptive field size, decreased irradiance threshold, and maintenance of the contrast threshold (Xia et al., 2015). Retinal dystrophy can be caused by defects in the phagocytosis by retinal pigment epithelium cells of the photoreceptor outer segments that are shed, a continuous and extremely active process involving thousands of shed membranous disks each day. TUDCA was found to increase phagocytic activity and inhibit hydrogen peroxide induced impairment of phagocytosis by both cultured ARPE-19 cells and primary human retinal pigment epithelium cells (Murase et al., 2015). The phosphorylation of MerTK was significantly increased by TUDCA in a concentration-dependent manner but did not affect expression of the ER stress marker glucose regulated protein-78 (GRP-78).

Bile acids also had an effect in ameliorating cell death in a model of retinal detachment via intraperitoneal injection of TUDCA at 3 and 5 days post detachment, times when acute photoreceptor loss occurs in the outer nuclear layer of the retina (Mantopoulos et al., 2011). TUDCA treatment also blocked the production of protein carbonyls with decreased caspase activation but did not decrease endoplasmic reticulum (ER) stress. In a diabetic retinopathy model, TUDCA significantly decreased apoptosis measured by TUNEL assay of primary rat retinal neural cells exposed to 30 mM glucose concentration, which causes caspase-independent cell death (Gaspar et al., 2013). TUDCA also decreased the amount of apoptosis-inducing factor (AIF) released from the mitochondria and its subsequent accumulation in the nucleus. Production of protein carbonyls and ROS were also significantly decreased after TUDCA treatment.

Over-stimulation of N-methyl-D-aspartate (NMDA) receptors, one of three ionotropic glutamate receptor subtypes that are expressed in inner retinal cells, by intravitreal injection of NMDA has frequently been used to model the cell death pathway that occurs in retinal diseases such as glaucoma (Danesh-Meyer, 2011). This model of excitotoxicity causes disruption of sodium-potassium balance, calcium overload, mitochondrial dysfunction, and oxidative stress (Gomez-Vicente et al., 2015). In mice, administration of TUDCA prior to the intravitreal injection of NMDA was found to increase survival of retinal ganglion cells.

## Acute stroke, spinal cord injury, and neuropathy

A single intravenous (i.v.) dose of TUDCA administered to rats 60 min after occlusion of the middle cerebral artery markedly reduced infarct size and apoptosis and preserved mitochondrial integrity (Rodrigues et al., 2002). The single i.v. dose resulted in a significant increase in brain UDCA levels from near undetectable levels to 0.15 nmol/g. TUDCA was also found to have an anti-inflammatory effect in the context of the central nervous system. TUDCA reduced glial cell activation and microglial chemotaxis and reduced expression of the MCP-1 chemoattractant and vascular adhesion proteins such as VCAM-1 in microglial cells and astrocytes treated with either interferon gamma (IFN-γ) or lipopolysaccharide and IFN-γ (Yanguas-Casás et al., 2014). Injection of TUDCA into the carotid artery 1 h prior to or up to 6 h after collagenase injection into the striatum to induce hemorrhagic lesions decreased lesion volumes, peri-hematoma apoptosis, and caspase activity at 2 days by about 50%, as well as decreased NF-κB and increased AKT activation that was associated with neurobehavioral improvement (Rodrigues et al., 2003). TUDCA injected intraperitoneally 1 min after spinal cord crush injury decreased apoptosis and corresponding tissue injury (Colak et al., 2008). In a phase II open-label study of oral doxycycline and TUDCA taken three times/day for 1 year in patients with transthyretin amyloidosis (ATTR), progression of neuropathy was stabilized (Obici et al., 2012).

## Modulation of neurotransmitter activity

The wake period of normal mice was found to be increased with administration of UDCA that also decreased slow wave sleep (Yanovsky et al., 2012). In contrast, administration of UDCA to histidine decarboxylase knockout mice, that are deficient in histamine that stimulates arousal, decreased wakefulness and altered cortical EEG and sleep-wake parameters. Using *in vitro* patch-clamp recordings from histaminergic neurons, UDCA was found to inhibit GABAergic currents and to serve as an antagonist for GABA_A_ receptors expressed in HEK293 cells. In one of the few studies to analyze more than one or two bile acids, a structure-function relationship analysis was performed using cultured hypothalamic neurons (Schubring et al., 2012). Bile acids were found to modulate firing frequency and synchrony, and to block activity of GABA_A_ and NMDA receptors. Antagonist activity for both the GABA_A_ and NMDA receptors was strongest for CDCA followed by DCA, CA, and dehydrocholate. Involvement of bile acid binding to TGR5 was excluded as a potential mechanism in the neurotransmitter receptor blockade (Keitel et al., 2010).

CA was found to serve as an inhibiter for N-type Ca^2+^ channel currents of neurons isolated from the caudal paravertebral sympathetic ganglia of adult bull frogs (*Rana catesbeiana*), although open and shut times, slope conductance, and single channel current amplitude, were not significantly affected (Lee et al., 2012). Overstimulation of glutamate receptors to induce excitotoxicity in neurons isolated from late stage fetal rat brains was largely suppressed by GUDCA. This bile acid appeared to suppress glutamate release in either normal or microglia-depleted hippocampal tissue slices (Silva et al., 2012). Pretreatment of primary cultures of fetal rat cortical neurons with TUDCA significantly reduced glutamate excitotoxicity associated cell death. TUDCA treatment resulted in phosphorylation and translocation of the pro-apoptotic Bad protein from the mitochondria to the cytosol. Inhibiting the PI3K signaling pathway blocked the anti-apoptotic effects of TUDCA (Castro et al., 2004).

## Potential as neurotrophic factors

Neurotrophic factors are classified primarily as proteins that bind to receptors, e.g., tropomyosin related kinase receptors TrkA, TrkB, and TrkC and p75 neurotrophin receptor (p75NTR) that can modulate Trk activation, to activate signaling pathways that promote neuronal growth and survival (Rodrigues et al., 2014). Several molecules have been identified as neurotrophic factors, including brain-derived neurotrophic factor (BDNF), ciliary neurotrophic factor (CNTF), and glial cell-line-derived neurotrophic factor (GDNF) that can stimulate the growth and differentiation of neuronal cells, as well as prevent cell death. Despite the therapeutic promise of such neurotrophic factors for treating neurodegenerative disorders (Tovar-Y-Romo et al., 2014), their limited bioavailability presents a major roadblock. Mimetics with more favorable bioavailability profiles have therefore been pursued (Tiwari and Chaturvedi, 2014), as well as investigation of proneurogenic peptides and small molecules that exhibit neuroprotective properties (Airavaara et al., 2012), including retinoic acid (Malik et al., 2000; Riancho et al., 2016). Retinoic acid shares a common role in regulating lipid homeostasis with bile acids, whose cognate receptor FXR can form heterodimers with RXRα. Thus, retinoic acid may activate the FXR signaling pathway and vice versa (Yang et al., 2014). Whether bile acids mediate effects in the nervous system through a similar mechanism is not known (Figure 1).

## Conclusions and outlook

**Figure 2:**
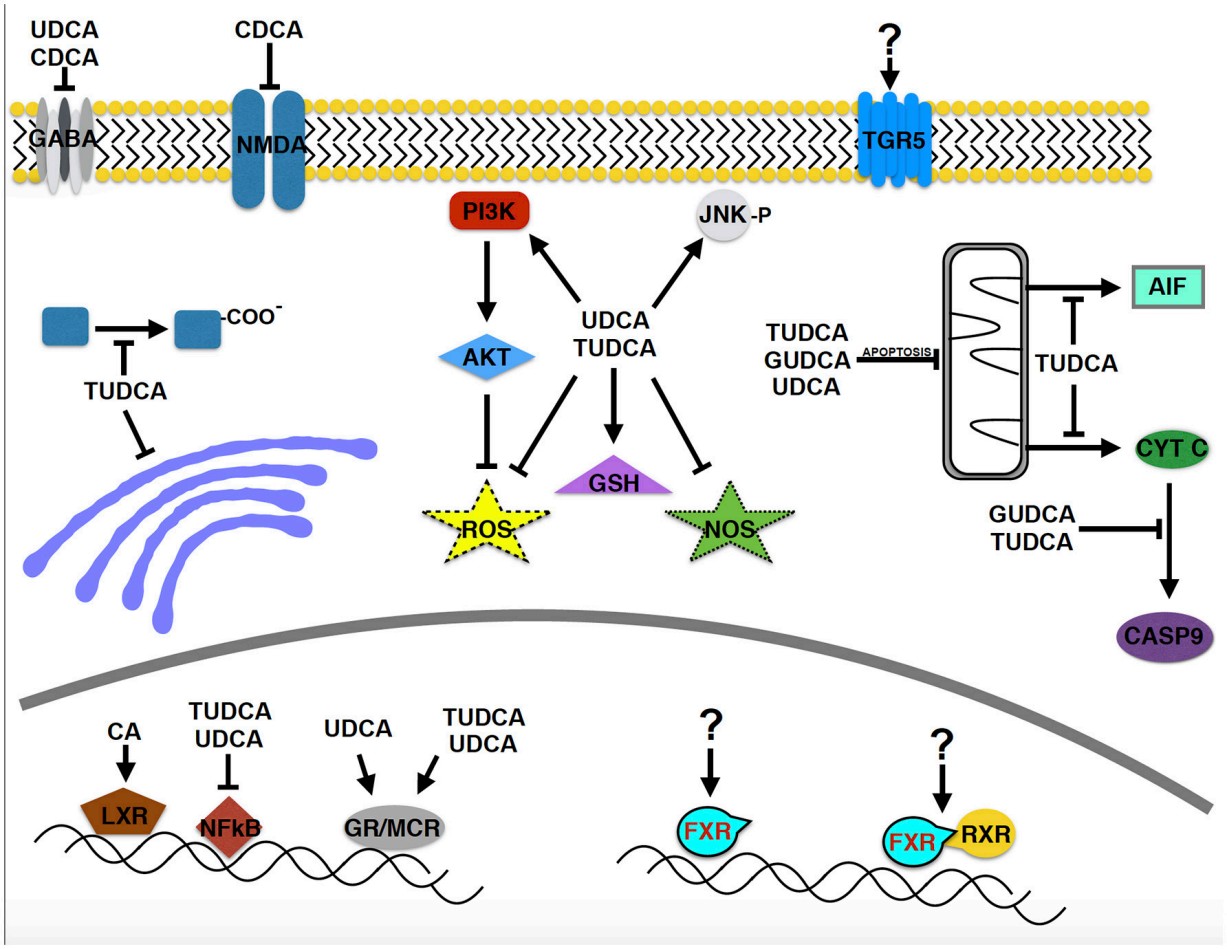
**Molecular pathways implicated in the neuroprotective effects of bile acids in neurodegenerative disease models**. Despite the relatively large structurally related group of bile acids, relatively few have been studied in neurodegenerative disorders. A major focus has been on apoptotic pathways and the PI3 kinase and AKT signaling pathway. Surprisingly, the primary signaling pathways through which bile acids act, TGR5, FXR, and FXR/RXR have received essentially no attention (designated by “?”), despite that retinoic acid is known to be a potent neurotrophic molecule.

Neuroprotective effects of several bile acids are well documented in a wide range of neurodegenerative diseases, including AD, PD, ALS, HD, and retinal degeneration, in cellular and animal models and in human clinical trials. Due to the historical precedent set by the use of bear bile in ancient Chinese medicine, the majority of the studies have focused on UDCA and its derivatives. However, bile acids are a relatively large group of structurally related molecules, thus little is known about the potential efficacy of other bile acid species or the roles of circulating vs. endogenous bile acids synthesized within the central nervous system. In addition, most of the mechanistic studies have been centered on apoptosis and related pathways (Figure 2). However, essentially no data is available on the primary signaling pathways through which bile acids act, the cellular receptor TGR5 and the nuclear receptors FXR and RXRα, despite the well-known function of retinoic acid as a potent neurotrophic molecule. Determining the precise molecular mechanism(s) of neuroprotection by bile acids in neurodegenerative disorders will be important to realize their future therapeutic potential.

## Author contributions

GG and HA designed, wrote, revised, and edited the manuscript. Both authors approved the submitted version of the manuscript.

## Funding

This work was funded by the Lewis Katz School of Medicine at Temple University Department of Medical Genetics and Molecular Biochemistry and the Joseph & Rebecca Goodfriend Endowed Chair in Genetics.

### Conflict of interest statement

The authors declare that the research was conducted in the absence of any commercial or financial relationships that could be construed as a potential conflict of interest.
